# Synthesis and structure of a new bulky bis­(alkoxide) ligand on a terphenyl platform

**DOI:** 10.1107/S2056989021013438

**Published:** 2022-01-01

**Authors:** Sudheer S. Kurup, Sandra Nasser, Cassandra L. Ward, Stanislav Groysman

**Affiliations:** a Department of Chemistry, Wayne State University, 5101 Cass Avenue, Detroit, Michigan 48202, USA; b Lumigen Instrument Center, Wayne State University, 5101 Cass Avenue, Detroit, Michigan 48202, USA

**Keywords:** crystal structure, alkoxide, chelating ligand, 3 *d* metals

## Abstract

A new sterically bulky chelating bis­(alkoxide) ligand 3,3′-([1,1′:4′,1′′-terphen­yl]-2,2′′-di­yl)bis­(2,2,4,4-tetra­methyl­pentan-3-ol), (H_2_[OO]^tBu^), was prepared in a two-step process as the di­chloro­methane monosolvate, C_36_H_50_O_2_·CH_2_Cl_2_. The first step is a Suzuki–Miyaura coupling reaction between 2-bromo­phenyl­boronic acid and 1,4-di­iodo­benzene. The resulting 2,2′′-di­bromo-1,1′:4′,1′′-terphenyl was reacted with ^*t*^BuLi and hexa­methyl­acetone to obtain the desired product. The crystal structure of H_2_[OO]^tBu^ revealed an *anti* conformation of the [CPh_2_(OH)] fragments relative to the central phenyl. Furthermore, the hydroxyl groups point away from each other. Likely because of this *anti*–*anti* conformation, the attempts to synthesize first-row transition-metal complexes with H_2_[OO]^tBu^ were not successful.

## Abstract 2

A new potentially chelating bulky bis­(alkoxide) exhibits *anti* conformation of the O-atom donors in the solid state and does not form transition-metal complexes.

## Chemical context

**Figure 1:**
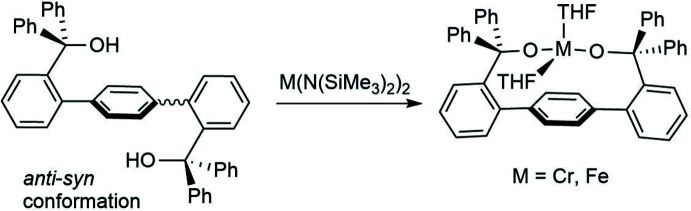
Schematic representation of the ‘*anti*–*syn’* structure of the previously synthesized H_2_[OO]^Ph^ ligand and its reactivity with transition-metal precursors.

Bulky alkoxides are becoming increasingly used as ancillary ligands in group-transfer chemistry and catalysis (Brazeau & Doerrer, 2019 ▸; Chua & Duong, 2014 ▸; Hannigan *et al.*, 2017 ▸; Jayasundara *et al.*, 2018 ▸; Wannipurage *et al.*, 2020 ▸). As a result of their stereoelectronic properties, profoundly weak-field bulky alkoxides enable formation of reactive low-coordinate high-spin middle and late transition-metal centers (Bellow *et al.*, 2016*b* ▸; Grass *et al.*, 2019*b* ▸). We have previously reported bulky monodentate alkoxides that led to reactive chromium and iron nitrene-transfer catalysts, (Bellow *et al.*, 2015 ▸; Wannipurage *et al.*, 2021 ▸; Yousif *et al.*, 2015 ▸, 2018 ▸) and a series of low-coordinate cobalt carbene complexes capable of carbene transfer to isocyanides (Bellow *et al.*, 2016*a* ▸; Grass *et al.*, 2019*a* ▸, 2020 ▸). However, the lability of monodentate alkoxides affected catalyst stability and the substrate scope. To remediate the problem of lability of monodentate alkoxides, we have designed and synthesized a new chelating bis(alkoxide) ligand [1,1′:4′,1′′-terphen­yl]-2,2′′-diylbis(di­phenyl­methanol) (H_2_[OO]^Ph^) (Fig. 1 ▸) (Kurup *et al.* 2019 ▸). The H_2_[OO]^Ph^ ligand employs a 1,1′:4′,1′′-terphenyl platform, which increases the bite angle between the alkoxide donors to form approximately seesaw transition-metal centers. While the isolated ligand precursor H_2_[OO]^Ph^ exhibits an *anti* conformation of the [CPh_2_(OH)] fragments relative to the central phenyl in the solid state (crystals obtained at 238 K), the hydroxyl groups point towards the central phenyl, exhib­iting overall an *anti–syn* conformation (Fig. 1 ▸) (Kurup *et al.*, 2019 ▸). Furthermore, while two different isomers were observed by ^1^H NMR spectroscopy at low temperatures, a single species was observed at room temperature, suggesting facile equilibration of *anti* and *syn* conformers. As a result, H_2_[OO]^Ph^ led to the formation of the desired bis­(alkoxide) complexes with iron and chromium (Fig. 1 ▸) (Kurup *et al.*, 2019 ▸, 2020 ▸). The resulting iron complex exhibited broader range of nitrene transfer reactivity, forming a variety of symmetric azoarenes. 
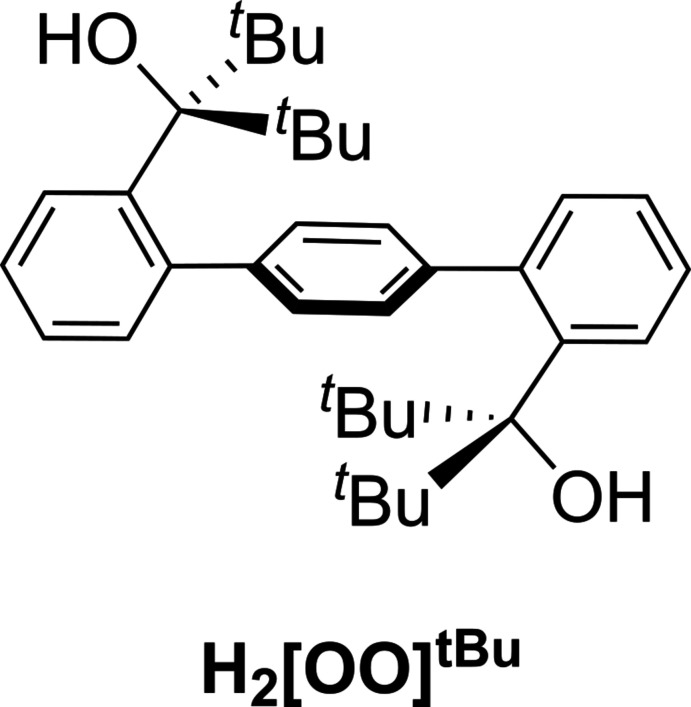


**Figure 2:**
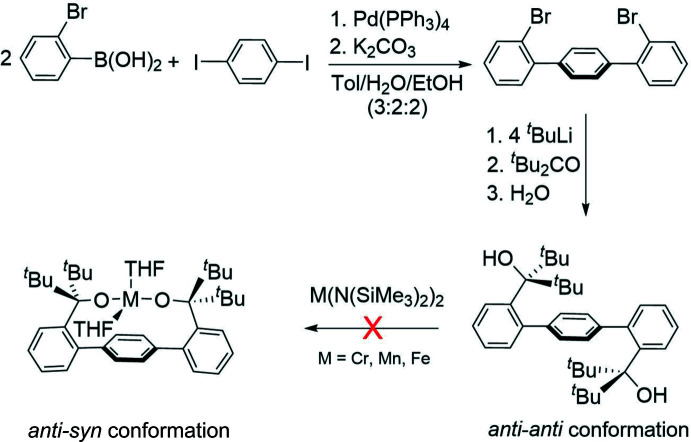
Synthesis of H_2_[OO]^tBu^, its schematic structure, and the lack of well-defined reactivity with transition-metal amide precursors.

The success of this strategy led us to design a new, even bulkier ligand (H_2_[OO]^tBu^). The ligand was synthesized in a two-step procedure as described in Fig. 2 ▸. Previously reported 2,2′′-di­bromo-1,1′:4′,1′′-terphenyl was synthesized through a Suzuki–Miyaura coupling reaction between 2-bromo­phenyl­boronic acid and 1,4-di­iodo­benzene following a literature procedure (Velian *et al.*, 2010 ▸). Next, 2,2′′-di­bromo-1,1′:4′,1′′-terphenyl was treated with ^*t*^BuLi followed by hexa­methyl­acetone. The formation of the desired product H_2_[OO]^tBu^ (35% isolated yield) was accompanied by the formation of significant amounts of *p*-terphenyl by-product (38% isolated yield). H_2_[OO]^tBu^ was characterized by ^1^H and ^13^C NMR spectroscopy, high-resolution mass spectrometry, and X-ray crystallography. ^1^H NMR spectroscopy demonstrates the presence of two isomers at room temperature in an approximately 2:1 ratio, as manifested by two *tert*-butyl resonances (1.05 and 1.03 ppm) and two OH resonances (2.09 and 2.07 ppm). This observation suggests that, in contrast to H_2_[OO]^Ph^, various isomers of H_2_[OO]^tBu^ do not readily inter­convert at room temperature, possibly due to the more significant steric hindrance of the *tert*-butyl groups. An X-ray crystallography study (see below) suggests that in at least one of these isomers the hydroxyl groups are pointing away from each other; such an isomer is unlikely to coordinate a single metal in a chelating fashion. Accordingly, the reaction of H_2_[OO]^tBu^ with several representative transition-metal amides (*M* = Cr, Mn, Fe) failed to produce isolable complexes.

## Structural commentary

**Figure 3:**
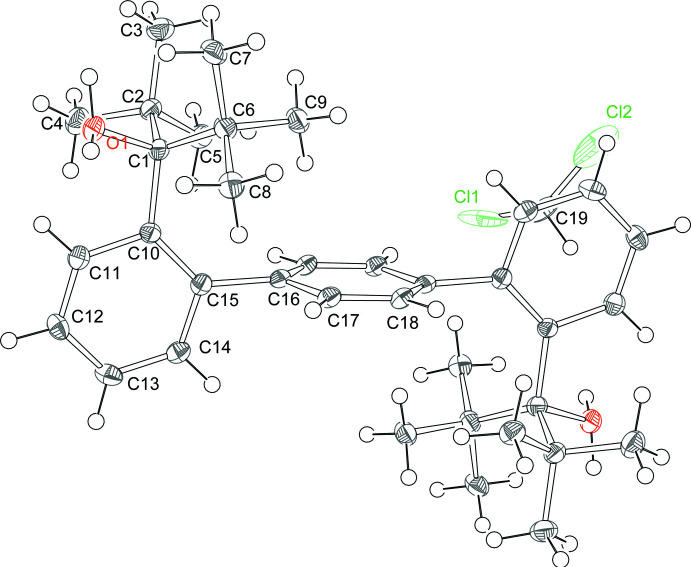
The structure of H_2_[OO]^tBu^ (50% probability ellipsoids) is shown with the co-crystallized di­chloro­methane solvent mol­ecule. The di­chloro­methane carbon atom was found to be disordered about an inversion center; only one orientation is shown, which is not the one belonging to the asymmetric unit. Hydroxyl H atoms are disordered over two positions, both positions are shown above.

The crystals of H_2_[OO]^tBu^ were obtained from di­chloro­methane at 238 K. The structure crystallized in space group *P* 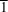 and is presented in Fig. 3 ▸. Selected bond distances and angles are given in Table 1 ▸. H_2_[OO]^tBu^ exhibits a crystallographic inversion center, with only half of the mol­ecule occupying the asymmetric unit. In addition to H_2_[OO]^tBu^, the structure contains one solvent mol­ecule (CH_2_Cl_2_) disordered by symmetry over two positions. Selected bond distances, angles, and torsion angles appear in Table 1 ▸. The lateral phenyls of the terphenyl unit are approximately perpendicular to the central phenyl ring, as indicated by the corresponding torsion angles close to 90° (see Table 1 ▸). Similar to the structure of H_2_[OO]^Ph^, H_2_[OO]^tBu^ manifests an *anti* conformation of the two ‘[C^t^Bu_2_(OH)]’ donors relative to the central phenyl ring. In contrast to the structure of H_2_[OO]^Ph^, the hydroxyls point away from each other in the structure of H_2_[OO]^tBu^, leading to an overall *anti*–*anti* conformation. This disposition results in the placement of the *tert*-butyl groups above and below the central phenyl ring. The presence of bulky groups on both sides of the central phenyl is likely responsible for the distortion of the terphenyl fragment, which is indicated by the C10—C15—C16 angle of 130.70 (15)° and the C14—C15—C16 angle of 110.28 (15)°. Same distortion is likely responsible for the slight variation in (lateral) phenyl bond distances (Table 1 ▸).

## Supra­molecular features

**Figure 4:**
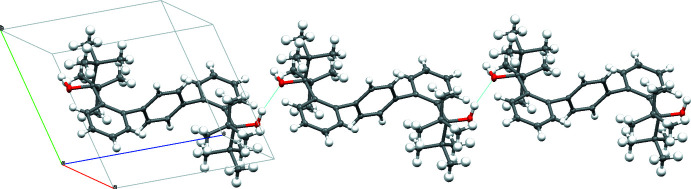
Chain of H_2_[OO]^tBu^ mol­ecules, bridged by hydrogen bonds (indicated in light blue).

H_2_[OO]^tBu^ forms one-dimensional polymer chains held together by hydrogen bonding between two neighboring mol­ecules (Table 2 ▸). One polymer chain is shown in Fig. 4 ▸. This chain-like structure results from the *anti*–*anti* conformation of H_2_[OO]^tBu^ in which both hydroxyl groups are pointing outward and thus can hydrogen bond with neighboring mol­ecules. The hydrogen-bond distance (indicated by the light-blue dashed lines in Fig. 4 ▸) is 2.13 (3) Å. It is also noted that, due to the inversion center present within the mol­ecule, the hydroxyl hydrogen atoms are disordered over two positions. As the diffraction data was of adequate quality, we were able to locate both hydrogen positions in the difference map. The corresponding O—H bonds are very similar, 0.93 (2) and 0.94 (2) Å. Only one of these hydrogen atoms participates in the hydrogen-bonding network (alternating conformations for consecutive mol­ecules). The solvent mol­ecules are positioned above and below the chains.

## Database survey

H_2_[OO]^tBu^ is a new compound that has not been previously synthesized and structurally characterized. As described above, the synthesis, structure, and coordination chemistry of the related compound H_2_[OO]^Ph^ has been previously reported by us (Kurup *et al.*, 2019 ▸) and reported in the Cambridge Structural Database (Groom *et al.*, 2016 ▸). We note that Agapie and coworkers have previously investigated structurally related 2,2′′-diphosphine-1,1′:4′,1′′-terphenyl ligands (Bailey & Agapie, 2021 ▸; Buss *et al.*, 2017 ▸) and Fortier and coworkers have investigated structurally related 2,2′′-di­amide-1,1′:4′,1′′-terphenyl ligands (Fortier *et al.*, 2017 ▸; Yadav *et al.*, 2020 ▸). In contrast to H_2_[OO]^tBu^, both the diphosphine and the di­amide terphenyl ligands serve as chelates for transition metals, adopting a *syn* geometry for the phosphine/amide donors relative to the central phenyl ring.

## Synthesis and crystallization

2,2′′-Di­bromo-1,1′:4′,1′′-terphenyl (Velian *et al.*, 2010 ▸) (1.00 g, 2.5 mmol) was dissolved in 30 mL THF and cooled under 238 K. To the cold solution ^*t*^BuLi (1.7 *M* in pentane, 6.4 mL, 10.8 mmol) was added dropwise and the resulting solution was stirred for 4 h. This reaction mixture was then transferred to a round-bottom flask containing hexa­methyl­acetone (8.7 ml, 5 mmol) in 20 mL of hexane and stirred for 24 h. The organic contents were extracted using a di­chloro­methane–water solvent system. The organic phase was dried over MgSO_4_ and filtered. The filtrate was concentrated using a rotatory evaporator. The desired product H_2_[OO]^tBu^ was separated in 35% yield (0.464 g, 0.9 mmol) by column chromatography on silica gel using 3% ethyl acetate in hexane. *para-T*erphenyl (1,1′:4′,1′′-terphen­yl) was found to be a major byproduct (38% yield, 0.504 g, 2.2 mmol). Purified H_2_[OO]^tBu^ was recrystallized from di­chloro­methane at 238 K to obtain colorless crystals suitable for X-ray crystallography. ^1^H NMR (298 K, 400 MHz, CD_2_Cl_2_) δ 1.05 (*s*, 25H, CH_3_) , 1.03 (*s*, 11H, CH_3_), 2.07 (*s*, 1H, OH), 2.09 (*s*, 1H, OH), 6.89 (*d*, *J* = 6.9 Hz, 1H, *ortho*-H), 6.96 (*d*, *J* = 7.5 Hz, 1H, *ortho*-H), 7.19 (*t*, *J* = 8.7 Hz, 2H, *para*-H), 7.33 (*m*, 6H), 8.28 (*d*, *J* = 8.2 Hz, 2H, *ortho*-H). ^13^C NMR (298 K, 125 MHz, CD_2_Cl_2_) δ 29.99, 30.28, 65.61, 87.36, 125.15, 126.08, 129.47, 130.19, 130.92, 131.37, 134.40, 134.84, 140.76, 144.07, 146.32. HRMS (*m*/*z*): Calculated [*M* - H]^+^ 515.39, found 515.36.

## Refinement

Crystal data, data collection and structure refinement details are summarized in Table 3 ▸. Data were acquired at 100 K with an Oxford 800 Cryostream low-temperature apparatus. Hydrogen atoms were placed in calculated positions using a standard riding model and refined isotropically (with the exception of hydroxyl hydrogens); all other atoms were refined anisotropically. The hydroxyl hydrogens were found to be disordered (due to the inversion center located at the hydrogen bond to the adjacent H_2_[OO]^tBu^) over two positions. Two alternating positions were identified from the difference-Fourier maps and refined to 50% occupancy. The CH_2_Cl_2_ solvent was also disordered by symmetry over two positions and refined with 50% occupancy.

## Supplementary Material

Crystal structure: contains datablock(s) I. DOI: 10.1107/S2056989021013438/jy2014sup1.cif

Structure factors: contains datablock(s) I. DOI: 10.1107/S2056989021013438/jy2014Isup2.hkl

Supporting information file. DOI: 10.1107/S2056989021013438/jy2014Isup3.cml

CCDC reference: 2129676

Additional supporting information:  crystallographic information; 3D view; checkCIF report

## Figures and Tables

**Table 1:** Selected bond distances (Å), angles, and torsion angles (°) in the structure of H_2_[OO]^tBu^

Selected bond distances			
O1—C1	1.451 (2)	C11—C12	1.387 (3)
C1—C2	1.591 (3)	C12—C13	1.371 (3)
C1—C6	1.591 (3)	C13—C14	1.380 (3)
C1—C10	1.586 (2)	C14—C15	1.393 (2)
C15—C16	1.511 (2)	C15—C10	1.426 (2)
C10—C11	1.408 (2)		
			
Selected bond angles			
O1—C1—C10	104.99 (13)	C1—C10—C11	113.90 (15)
O1—C1—C2	104.30 (13)	C1—C10—C15	130.84 (15)
O1—C1—C6	104.28 (13)	C14—C15—C10	119.01 (16)
C10—C1—C2	108.49 (14)	C16—C15—C14	110.28 (15)
			
Selected torsion angles			
C14—C15—C16—C17	−84.3 (2)	C14—C15—C16—C18	86.4 (2)
C10—C15—C16—C17	96.7 (2)	C10—C15—C16—C18	−92.7 (2)

**Table 2:** Hydrogen-bond geometry (Å, °)

*D*—H⋯*A*	*D*—H	H⋯*A*	*D*⋯*A*	*D*—H⋯*A*
O1—H1*A*⋯O1^i^	0.93 (4)	2.13 (3)	3.0066 (19)	157 (4)

Symmetry code: (i) -x+1, -y+1, -z.

**Table 3:** Experimental details

Crystal data	
Chemical formula	C_36_H_50_O_2_·CH_2_Cl_2_
*M*_r_	599.68
Crystal system, space group	Triclinic, *P*\overline{1}
Temperature (K)	100
*a*, *b*, *c* (Å)	8.2449 (4), 9.1248 (4), 12.1825 (6)
α, β, γ (°)	101.530 (2), 102.729 (3), 109.200 (2)
*V* (Å^3^)	806.53 (7)
*Z*	1
Radiation type	Mo *K*α
μ (mm^−1^)	0.23
Crystal size (mm)	0.15 × 0.1 × 0.04

Data collection	
Diffractometer	Bruker APEXII CCD
Absorption correction	Multi-scan (*SADABS*; Krause *et al.*, 2015 ▸)
*T*_min_, *T*_max_	0.722, 0.746
No. of measured, independent and observed [*I* > 2σ(*I*)] reflections	27197, 3559, 2769
*R*_int_	0.037
(sin θ/λ)_max_ (Å^−1^)	0.644

Refinement	
*R*[*F* ^2^ > 2σ(*F* ^2^)], *wR*(*F* ^2^), *S*	0.051, 0.136, 1.05
No. of reflections	3559
No. of parameters	211
No. of restraints	27
H-atom treatment	H atoms treated by a mixture of independent and constrained refinement
Δρ_max_, Δρ_min_ (e Å^−3^)	0.57, −0.43

Computer programs: *APEX2* and *SAINT* (Bruker, 2016 ▸), *SHELXT2018/2* (Sheldrick, 2015*a* ▸), *SHELXL2018/3* (Sheldrick, 2015*b* ▸), and *OLEX2* (Dolomanov *et al.*, 2009 ▸).

## supplementary crystallographic information

### Crystal data

**Table:**

C_36_H_50_O_2_·CH_2_Cl_2_	*Z* = 1
*M**_r_* = 599.68	*F*(000) = 324
Triclinic, *P*1	*D*_x_ = 1.235 Mg m^−^^3^
*a* = 8.2449 (4) Å	Mo *K*α radiation, λ = 0.71073 Å
*b* = 9.1248 (4) Å	Cell parameters from 9980 reflections
*c* = 12.1825 (6) Å	θ = 2.5–27.2°
α = 101.530 (2)°	µ = 0.23 mm^−^^1^
β = 102.729 (3)°	*T* = 100 K
γ = 109.200 (2)°	Prism, colourless
*V* = 806.53 (7) Å^3^	0.15 × 0.1 × 0.04 mm

### Data collection

**Table:**

Bruker APEXII CCD diffractometer	3559 independent reflections
Radiation source: sealed tube	2769 reflections with *I* > 2σ(*I*)
Graphite monochromator	*R*_int_ = 0.037
Detector resolution: 8 pixels mm^-1^	θ_max_ = 27.2°, θ_min_ = 1.8°
ω and φ scans	*h* = −10→10
Absorption correction: multi-scan (SADABS; Krause *et al.*, 2015)	*k* = −11→11
*T*_min_ = 0.722, *T*_max_ = 0.746	*l* = −15→15
27197 measured reflections	

### Refinement

**Table:**

Refinement on *F*^2^	Primary atom site location: dual
Least-squares matrix: full	Hydrogen site location: mixed
*R*[*F*^2^ > 2σ(*F*^2^)] = 0.051	H atoms treated by a mixture of independent and constrained refinement
*wR*(*F*^2^) = 0.136	*w* = 1/[σ^2^(*F*_o_^2^) + (0.0594*P*)^2^ + 0.6108*P*] where *P* = (*F*_o_^2^ + 2*F*_c_^2^)/3
*S* = 1.05	(Δ/σ)_max_ < 0.001
3559 reflections	Δρ_max_ = 0.57 e Å^−^^3^
211 parameters	Δρ_min_ = −0.43 e Å^−^^3^
27 restraints	

### Special details

**Table:**

Geometry. All esds (except the esd in the dihedral angle between two l.s. planes) are estimated using the full covariance matrix. The cell esds are taken into account individually in the estimation of esds in distances, angles and torsion angles; correlations between esds in cell parameters are only used when they are defined by crystal symmetry. An approximate (isotropic) treatment of cell esds is used for estimating esds involving l.s. planes.
Refinement. Used Part -1 on the dichloromethane (DCM) and hydroxyl hydrogens because they were disordered by inverison symmetry (sof=0.5). In addition, RIGU/DFIX/SIMU were employed to model the disorder of the DCM solvent.The structure of H2[OO]tBu was collected on a Bruker X8 APEX-II diffractometer with MoKα radiation and a graphite monochromator. The diffraction intensities were measured using a Bruker APEX-II CCD detector. Data were acquired at 100?K with an Oxford 800 Cryostream low-temperature apparatus. The data were processed using APEX3 software supplied by Bruker AXS. The structures were solved by Intrinsic Phasing using SHELXT (Sheldrick, 2015a) and refined with SHELXL-2018 (Sheldrick 2015b) using Olex2 (Dolomanov, 2009).

### Fractional atomic coordinates and isotropic or equivalent isotropic displacement parameters (Å^2^)

**Table:**

	*x*	*y*	*z*	*U*_iso_*/*U*_eq_	Occ. (<1)
O1	0.55932 (17)	0.63933 (16)	0.10947 (11)	0.0224 (3)	
H1A	0.556 (6)	0.555 (4)	0.050 (3)	0.027*	0.5
H1B	0.594 (6)	0.737 (3)	0.088 (4)	0.027*	0.5
C17	0.8826 (2)	0.5620 (2)	0.53594 (15)	0.0176 (4)	
H17	0.801780	0.603330	0.562488	0.021*	
C1	0.6733 (2)	0.6559 (2)	0.22428 (15)	0.0174 (4)	
C15	0.6438 (2)	0.4210 (2)	0.33668 (15)	0.0166 (4)	
C14	0.5253 (2)	0.2805 (2)	0.34915 (16)	0.0200 (4)	
H14	0.571142	0.235101	0.406337	0.024*	
C16	0.8316 (2)	0.4698 (2)	0.41871 (15)	0.0168 (4)	
C18	1.0488 (2)	0.5946 (2)	0.61443 (15)	0.0179 (4)	
H18	1.081840	0.662282	0.692598	0.021*	
C10	0.5770 (2)	0.4932 (2)	0.25311 (15)	0.0164 (4)	
C12	0.2780 (2)	0.2706 (2)	0.20251 (16)	0.0218 (4)	
H12	0.154405	0.221280	0.156261	0.026*	
C8	0.8541 (3)	0.5146 (2)	0.13697 (16)	0.0228 (4)	
H8A	0.823600	0.436332	0.181305	0.034*	
H8B	0.971115	0.526787	0.125134	0.034*	
H8C	0.760907	0.475262	0.060292	0.034*	
C13	0.3453 (3)	0.2034 (2)	0.28338 (16)	0.0233 (4)	
H13	0.270041	0.106475	0.293847	0.028*	
C2	0.6571 (2)	0.8019 (2)	0.31128 (16)	0.0196 (4)	
C11	0.3922 (2)	0.4115 (2)	0.18886 (16)	0.0201 (4)	
H11	0.342427	0.455795	0.132331	0.024*	
C9	1.0251 (2)	0.7558 (2)	0.32004 (16)	0.0230 (4)	
H9A	1.046663	0.870258	0.352693	0.034*	
H9B	1.133028	0.748183	0.302648	0.034*	
H9C	0.997886	0.697588	0.377407	0.034*	
C6	0.8648 (2)	0.6799 (2)	0.20637 (16)	0.0205 (4)	
C7	0.9138 (3)	0.7914 (2)	0.12807 (17)	0.0242 (4)	
H7A	0.818087	0.748131	0.052235	0.036*	
H7B	1.028136	0.795658	0.114886	0.036*	
H7C	0.926383	0.901068	0.167499	0.036*	
C5	0.7299 (3)	0.8151 (2)	0.44128 (16)	0.0230 (4)	
H5A	0.660312	0.715436	0.456406	0.035*	
H5B	0.718633	0.908189	0.490347	0.035*	
H5C	0.857219	0.830149	0.460379	0.035*	
C3	0.7517 (3)	0.9677 (2)	0.29460 (18)	0.0263 (4)	
H3A	0.882351	0.996007	0.317126	0.039*	
H3B	0.726025	1.050519	0.344213	0.039*	
H3C	0.707206	0.962474	0.211807	0.039*	
C4	0.4579 (3)	0.7765 (2)	0.29020 (18)	0.0264 (4)	
H4A	0.403414	0.768737	0.208073	0.040*	
H4B	0.449706	0.868637	0.342607	0.040*	
H4C	0.393527	0.676084	0.306465	0.040*	
Cl1	0.3212 (4)	−0.0907 (3)	−0.0033 (2)	0.0621 (10)	0.5
Cl2	0.6911 (5)	0.0667 (4)	−0.0006 (3)	0.0715 (11)	0.5
C19	0.5047 (6)	0.0957 (5)	0.0345 (3)	0.0311 (10)	0.5
H19A	0.539953	0.150232	0.119918	0.037*	0.5
H19B	0.468532	0.166861	−0.008572	0.037*	0.5

### Atomic displacement parameters (Å^2^)

**Table:**

	*U*^11^	*U*^22^	*U*^33^	*U*^12^	*U*^13^	*U*^23^
O1	0.0208 (7)	0.0255 (7)	0.0181 (7)	0.0073 (6)	0.0002 (5)	0.0097 (6)
C17	0.0209 (9)	0.0168 (9)	0.0187 (9)	0.0109 (7)	0.0066 (7)	0.0063 (7)
C1	0.0182 (9)	0.0162 (9)	0.0145 (8)	0.0053 (7)	0.0007 (7)	0.0045 (7)
C15	0.0183 (9)	0.0159 (8)	0.0151 (8)	0.0078 (7)	0.0039 (7)	0.0028 (7)
C14	0.0243 (9)	0.0202 (9)	0.0181 (9)	0.0105 (8)	0.0067 (7)	0.0079 (7)
C16	0.0186 (9)	0.0149 (8)	0.0175 (8)	0.0065 (7)	0.0042 (7)	0.0077 (7)
C18	0.0224 (9)	0.0155 (8)	0.0150 (8)	0.0081 (7)	0.0036 (7)	0.0044 (7)
C10	0.0169 (8)	0.0143 (8)	0.0166 (8)	0.0058 (7)	0.0040 (7)	0.0036 (7)
C12	0.0172 (9)	0.0220 (9)	0.0183 (9)	0.0030 (7)	0.0014 (7)	0.0018 (7)
C8	0.0234 (9)	0.0247 (10)	0.0207 (9)	0.0093 (8)	0.0073 (8)	0.0070 (8)
C13	0.0248 (10)	0.0181 (9)	0.0237 (10)	0.0028 (8)	0.0093 (8)	0.0069 (8)
C2	0.0205 (9)	0.0171 (9)	0.0202 (9)	0.0082 (7)	0.0027 (7)	0.0058 (7)
C11	0.0198 (9)	0.0216 (9)	0.0176 (9)	0.0077 (7)	0.0030 (7)	0.0065 (7)
C9	0.0181 (9)	0.0232 (10)	0.0225 (9)	0.0041 (8)	0.0035 (7)	0.0060 (8)
C6	0.0178 (9)	0.0224 (9)	0.0200 (9)	0.0057 (7)	0.0050 (7)	0.0077 (7)
C7	0.0212 (9)	0.0246 (10)	0.0217 (9)	0.0022 (8)	0.0051 (7)	0.0092 (8)
C5	0.0271 (10)	0.0201 (9)	0.0198 (9)	0.0099 (8)	0.0042 (8)	0.0037 (7)
C3	0.0308 (11)	0.0181 (9)	0.0281 (10)	0.0092 (8)	0.0059 (8)	0.0071 (8)
C4	0.0260 (10)	0.0268 (10)	0.0287 (10)	0.0153 (8)	0.0065 (8)	0.0066 (8)
Cl1	0.103 (2)	0.0143 (6)	0.0325 (10)	−0.0108 (9)	0.0021 (11)	0.0079 (6)
Cl2	0.120 (2)	0.104 (2)	0.0643 (16)	0.093 (2)	0.0660 (15)	0.0541 (14)
C19	0.057 (3)	0.029 (2)	0.0190 (19)	0.030 (2)	0.0121 (19)	0.0086 (17)

### Geometric parameters (Å, º)

**Table:**

O1—H1A	0.933 (19)	C2—C5	1.530 (2)
O1—H1B	0.944 (19)	C2—C3	1.535 (3)
O1—C1	1.451 (2)	C2—C4	1.535 (3)
C17—H17	0.9500	C11—H11	0.9500
C17—C16	1.395 (2)	C9—H9A	0.9800
C17—C18	1.386 (2)	C9—H9B	0.9800
C1—C10	1.586 (2)	C9—H9C	0.9800
C1—C2	1.591 (3)	C9—C6	1.533 (2)
C1—C6	1.591 (3)	C6—C7	1.548 (2)
C15—C14	1.393 (2)	C7—H7A	0.9800
C15—C16	1.511 (2)	C7—H7B	0.9800
C15—C10	1.426 (2)	C7—H7C	0.9800
C14—H14	0.9500	C5—H5A	0.9800
C14—C13	1.380 (3)	C5—H5B	0.9800
C16—C18^i^	1.393 (3)	C5—H5C	0.9800
C18—H18	0.9500	C3—H3A	0.9800
C10—C11	1.408 (2)	C3—H3B	0.9800
C12—H12	0.9500	C3—H3C	0.9800
C12—C13	1.371 (3)	C4—H4A	0.9800
C12—C11	1.387 (3)	C4—H4B	0.9800
C8—H8A	0.9800	C4—H4C	0.9800
C8—H8B	0.9800	Cl1—C19	1.755 (4)
C8—H8C	0.9800	Cl2—C19	1.768 (4)
C8—C6	1.539 (3)	C19—H19A	0.9900
C13—H13	0.9500	C19—H19B	0.9900
			
H1A—O1—H1B	109 (4)	C10—C11—H11	117.8
C1—O1—H1A	113 (3)	C12—C11—C10	124.45 (17)
C1—O1—H1B	112 (3)	C12—C11—H11	117.8
C16—C17—H17	119.3	H9A—C9—H9B	109.5
C18—C17—H17	119.3	H9A—C9—H9C	109.5
C18—C17—C16	121.35 (16)	H9B—C9—H9C	109.5
O1—C1—C10	104.99 (13)	C6—C9—H9A	109.5
O1—C1—C2	104.30 (13)	C6—C9—H9B	109.5
O1—C1—C6	104.28 (13)	C6—C9—H9C	109.5
C10—C1—C2	108.49 (14)	C8—C6—C1	108.22 (14)
C10—C1—C6	115.07 (14)	C8—C6—C7	104.41 (15)
C6—C1—C2	118.18 (14)	C9—C6—C1	115.03 (15)
C14—C15—C16	110.28 (15)	C9—C6—C8	111.07 (15)
C14—C15—C10	119.01 (16)	C9—C6—C7	104.92 (15)
C10—C15—C16	130.70 (15)	C7—C6—C1	112.70 (15)
C15—C14—H14	118.1	C6—C7—H7A	109.5
C13—C14—C15	123.88 (17)	C6—C7—H7B	109.5
C13—C14—H14	118.1	C6—C7—H7C	109.5
C17—C16—C15	121.05 (15)	H7A—C7—H7B	109.5
C18^i^—C16—C17	117.09 (16)	H7A—C7—H7C	109.5
C18^i^—C16—C15	121.23 (15)	H7B—C7—H7C	109.5
C17—C18—C16^i^	121.49 (16)	C2—C5—H5A	109.5
C17—C18—H18	119.3	C2—C5—H5B	109.5
C16^i^—C18—H18	119.3	C2—C5—H5C	109.5
C15—C10—C1	130.84 (15)	H5A—C5—H5B	109.5
C11—C10—C1	113.90 (15)	H5A—C5—H5C	109.5
C11—C10—C15	115.18 (15)	H5B—C5—H5C	109.5
C13—C12—H12	120.3	C2—C3—H3A	109.5
C13—C12—C11	119.34 (17)	C2—C3—H3B	109.5
C11—C12—H12	120.3	C2—C3—H3C	109.5
H8A—C8—H8B	109.5	H3A—C3—H3B	109.5
H8A—C8—H8C	109.5	H3A—C3—H3C	109.5
H8B—C8—H8C	109.5	H3B—C3—H3C	109.5
C6—C8—H8A	109.5	C2—C4—H4A	109.5
C6—C8—H8B	109.5	C2—C4—H4B	109.5
C6—C8—H8C	109.5	C2—C4—H4C	109.5
C14—C13—H13	120.9	H4A—C4—H4B	109.5
C12—C13—C14	118.13 (17)	H4A—C4—H4C	109.5
C12—C13—H13	120.9	H4B—C4—H4C	109.5
C5—C2—C1	113.25 (14)	Cl1—C19—Cl2	110.9 (2)
C5—C2—C3	107.88 (15)	Cl1—C19—H19A	109.5
C5—C2—C4	105.36 (15)	Cl1—C19—H19B	109.5
C3—C2—C1	113.32 (15)	Cl2—C19—H19A	109.5
C3—C2—C4	106.35 (15)	Cl2—C19—H19B	109.5
C4—C2—C1	110.16 (15)	H19A—C19—H19B	108.1
			
O1—C1—C10—C15	162.41 (17)	C10—C1—C2—C5	54.70 (19)
O1—C1—C10—C11	−21.10 (19)	C10—C1—C2—C3	178.00 (14)
O1—C1—C2—C5	166.21 (14)	C10—C1—C2—C4	−63.00 (18)
O1—C1—C2—C3	−70.49 (18)	C10—C1—C6—C8	39.81 (19)
O1—C1—C2—C4	48.51 (18)	C10—C1—C6—C9	−85.03 (19)
O1—C1—C6—C8	−74.63 (16)	C10—C1—C6—C7	154.74 (15)
O1—C1—C6—C9	160.53 (15)	C10—C15—C14—C13	1.3 (3)
O1—C1—C6—C7	40.31 (19)	C10—C15—C16—C17	96.7 (2)
C1—C10—C11—C12	−177.15 (17)	C10—C15—C16—C18^i^	−92.7 (2)
C15—C14—C13—C12	−1.3 (3)	C13—C12—C11—C10	0.1 (3)
C15—C10—C11—C12	−0.1 (3)	C2—C1—C10—C15	−86.5 (2)
C14—C15—C16—C17	−84.3 (2)	C2—C1—C10—C11	89.94 (17)
C14—C15—C16—C18^i^	86.4 (2)	C2—C1—C6—C8	170.19 (14)
C14—C15—C10—C1	175.90 (17)	C2—C1—C6—C9	45.3 (2)
C14—C15—C10—C11	−0.6 (2)	C2—C1—C6—C7	−74.88 (19)
C16—C17—C18—C16^i^	−3.1 (3)	C11—C12—C13—C14	0.6 (3)
C16—C15—C14—C13	−177.91 (17)	C6—C1—C10—C15	48.4 (2)
C16—C15—C10—C1	−5.1 (3)	C6—C1—C10—C11	−135.13 (16)
C16—C15—C10—C11	178.46 (17)	C6—C1—C2—C5	−78.62 (19)
C18—C17—C16—C15	174.02 (16)	C6—C1—C2—C3	44.7 (2)
C18—C17—C16—C18^i^	3.0 (3)	C6—C1—C2—C4	163.68 (15)

Symmetry code: (i) −*x*+2, −*y*+1, −*z*+1.

### Hydrogen-bond geometry (Å, º)

**Table:**

*D*—H···*A*	*D*—H	H···*A*	*D*···*A*	*D*—H···*A*
O1—H1*A*···O1^ii^	0.93 (4)	2.13 (3)	3.0066 (19)	157 (4)

Symmetry code: (ii) −*x*+1, −*y*+1, −*z*.
